# Predictive modeling of postoperative gastrointestinal dysfunction: the role of serum bilirubin, sodium levels, and surgical duration in gynecological cancer care

**DOI:** 10.1186/s12905-023-02779-1

**Published:** 2023-11-13

**Authors:** Lijuan He, Jun Hu, Yun Han, Wenli Xiong

**Affiliations:** 1 Health Management Center, Affiliated Hospital of Southwest Medical University, Luzhou, Sichuan 646000 People’s Republic of China; 2 The Department of Gynecology, Affiliated Hospital of Southwest Medical University, Luzhou, Sichuan 646000 People’s Republic of China; 3 Department of Urology, Yibin Fifth People’s Hospital, Yibin, Sichuan 644100 People’s Republic of China

**Keywords:** Gynecological Cancer, Postoperative gastrointestinal dysfunction, Predictive factors, Serum bilirubin, Sodium levels, Surgical duration, Personalized care

## Abstract

**Objective:**

To elucidate the role of preoperative serum bilirubin and sodium levels, along with the duration of surgery, in predicting postoperative gastrointestinal dysfunction (POGD) following gynecological cancer surgery, informing tailored perioperative strategies.

**Methods:**

We conducted a retrospective analysis of 281 patients undergoing gynecological cancer surgery between 2018 and 2023. This analysis focused on preoperative serum bilirubin and sodium levels and intraoperative factors (surgical duration) as potential predictors of POGD. Logistic regression models were utilized for analysis, controlling for relevant confounders.

**Results:**

Elevated preoperative serum bilirubin was associated with a reduced risk of POGD (mean level in non-POGD cases: 14.172 ± 4.0701, vs. POGD cases: 9.6429 ± 3.5351; *p* <  0.001), suggesting a protective role. Lower preoperative sodium levels were identified in the POGD group (136.26 mEq/L [IQR: 135.2–137.63]) compared to the non-POGD group (139.32 mEq/L [IQR: 137.7–140.75]; *p* <  0.001), highlighting its predictive value. Additionally, longer surgical duration was associated with increased POGD incidence, with POGD cases experiencing surgeries lasting 6.1547 ± 1.9426 hours compared to 4.5959 ± 1.5475 hours in non-POGD cases (*p* <  0.001).

**Conclusion:**

Our findings underscore the importance of serum bilirubin, sodium levels, and surgical duration as significant predictors of POGD in patients undergoing gynecological cancer surgery. These indicators should be integrated into a predictive model, aiding clinicians in identifying high-risk patients, allowing for personalized perioperative care adjustments, potentially mitigating POGD risks.

**Supplementary Information:**

The online version contains supplementary material available at 10.1186/s12905-023-02779-1.

## Introduction

Gynecological malignancies, encompassing cervical, ovarian, and endometrial cancers, remain a significant health burden, contributing markedly to morbidity and mortality among women globally [1]. Surgical interventions, although crucial for managing these malignancies, often lead to a range of postoperative complications. Postoperative Gastrointestinal Dysfunction (POGD) emerges as a predominant concern, affecting approximately 20% of gynecological cancer patients, thereby emphasizing the urgency of addressing this issue [2, 3].

POGD encompasses symptoms like nausea, vomiting, abdominal distention, delayed passage of flatus or stool, and prolonged inability to tolerate oral intake [4]. The incidence of postoperative nausea can reach up to 50%, vomiting about 30%, and paralytic ileus between 12.9 and 32%, significantly impacting postoperative recovery, prolonging hospital stays, and escalating healthcare costs [5]. Moreover, POGD substantially impairs the quality of life of affected individuals [6]. While extant literature has shed light on demographic factors such as age and Body Mass Index (BMI) as potential precursors to POGD [7], a notable research gap persists in understanding the collective impact of intraoperative, biochemical, and pre-existing conditions on POGD onset [8]. Enhanced Recovery After Surgery (ERAS) protocols have shown promise in improving postoperative gastrointestinal function, yet the comprehensive understanding and implementation remain in infancy, necessitating further investigation [9].

Serum bilirubin, apart from its conventional role as a hepatic function and hemolysis marker, has been associated with antioxidative and anti-inflammatory properties, potentially ameliorating gastrointestinal dysfunction post-surgery [10]. Sodium, a pivotal electrolyte, governs fluid balance and may have indirect ramifications for gastrointestinal motility, especially in the postoperative setting [11]. The surgical time and anesthesia duration are well-acknowledged for their bearing on postoperative outcomes, where extended surgical time may elevate the risk of complications like surgical site infections and venous thromboembolism, potentially delaying gastrointestinal recovery [12]. The type and duration of anesthesia can significantly affect the motility and function of the gut [13].

Amidst this backdrop, our study chiefly addresses the critical research query: What are the predictive factors for POGD in gynecological cancer patients, and how can a comprehensive model incorporating these factors be developed to predict POGD risk more accurately? Identifying this lacuna in the current scientific discourse, the primary objective of our study is twofold: to meticulously discern the predictive factors for POGD in patients who have undergone gynecological cancer surgeries, and to develop a comprehensive predictive model that encompasses these factors [14]. This endeavor aims to refine postoperative patient management, thereby reducing the associated risks of POGD [15].

In this manuscript, we delineate the findings of an exhaustive case-control study, representing, to our understanding, the inaugural concerted effort to evaluate these specific parameters as plausible precursors for POGD in the milieu of gynecological cancer patients. By elucidating these aspects, we aspire to pave the way for tailored perioperative care strategies, minimizing the incidence of POGD in this vulnerable demographic.

## Materials and methods

### Patients

Our study adheres to a retrospective design, molded by methodologies delineated in previous research, particularly the studies conducted by Onwude JL [16] and Chao X et al. [17]. We meticulously reviewed the medical records of 281 patients diagnosed with endometrial cancer, cervical cancer, or ovarian cancer who underwent surgical interventions at our institution between the years 2018 and 2023. These patients were identified from a comprehensive database maintained by our hospital, ensuring a robust data collection process. The surgical procedures encompassed extensive total hysterectomy, total hysterectomy ± bilateral adnexectomy, and supplementary procedures like pelvic lymph node dissection ± para-aortic lymph node dissection, conforming to standard surgical treatments for the mentioned cancers.

### Inclusion and exclusion criteria

The inclusion criteria were stringent, encompassing patients diagnosed with the aforementioned cancers and who underwent the specified surgical interventions. This criterion aimed to engender a cohort uniform concerning both the disease and the surgical intervention, thus maintaining a consistent surgical context for assessing POGD. Exclusion criteria were judiciously defined to mitigate potential confounding factors:Presence of thyroid disease, gastritis, or chronic constipation (defined as ≤2 bowel movements per week), as these conditions could inherently alter gastrointestinal motility and function, which could confound the POGD assessment;History of gastrointestinal surgery or prior treatment with pelvic abdominal radiotherapy or chemotherapy, as these interventions might intrinsically elevate the risk of POGD due to their direct effects on the gastrointestinal system;Undergoing intestinal anastomosis surgery or concurrent upper abdominal multi-organ surgery, known for their higher inherent risk of POGD due to direct manipulation of the gastrointestinal tract;Having a gastrointestinal decompression tube kept in place until 24 hours postoperatively, which could mask or alter the presentation of POGD symptoms. These exclusions were meticulously crafted to obviate potential confounders, ensuring that any identified risk factors are predominantly associated with the surgical process rather than pre-existing conditions.

Postoperatively, all eligible patients were diligently monitored for a week to discern the development of postoperative gastrointestinal dysfunction. This monitoring encompassed clinical evaluations, meticulously noting symptoms such as nausea, vomiting, abdominal distention, and assessing the patient’s ability to tolerate oral intake. The diagnostic criteria for POGD were predicated on a confluence of these clinical symptoms and were confirmed if a patient necessitated interventions like the administration of prokinetic agents or nasogastric tube insertion. Subsequently, patients were bifurcated into two groups: the case group (those who developed POGD) and the control group (those who did not), thereby showcasing the real-world incidence of POGD in our cohort.

### Variables

The data collection encompassed an array of variables segregated into distinct categories to enable a structured analysis and ensure a comprehensive understanding of the factors influencing postoperative gastrointestinal dysfunction (POGD). Demographic variables included age, gender, body mass index (BMI), and comorbidities, pivotal in understanding the baseline health status and potential risk factors for POGD. Preoperative laboratory examinations such as serum bilirubin, liver enzymes, creatinine, and electrolytes were collected, offering insights into the patients’ preoperative systemic health and potential susceptibility to POGD.

Anesthesia-related indicators, including the type of anesthesia, duration of anesthesia, and any anesthesia-related complications, were recorded given their potential influence on postoperative recovery and gastrointestinal function. Surgery-related indicators encompassed the type of surgery, duration of surgery, intraoperative blood loss, and any surgical complications, quintessential for understanding the surgical impact on postoperative gastrointestinal function. Information regarding the use of analgesic pumps, type of analgesics used, and duration of analgesic use were documented to assess the impact of pain management strategies on postoperative gastrointestinal recovery.

Additionally, other pertinent variables such as previous abdominal surgeries, perioperative medications, and postoperative complications were considered, as they could have a bearing on the development of POGD. In the statistical analyses, multicollinearity was meticulously reviewed among variables like ‘body mass index’ and ‘previous abdominal surgeries’. The variable ‘previous abdominal surgeries’ was identified to be collinear with ‘surgical duration’ and was thus omitted from the multivariate regression model to uphold the model’s integrity.

Handling of missing data was executed using multiple imputations to ensure a robust analysis, providing a more accurate estimation of missing values. Sensitivity analyses were conducted to affirm the robustness of our results, even when excluding patients with imputed data, thereby ensuring the reliability and validity of our findings. This structured approach in variable selection and handling missing data aims to provide a rigorous analytical framework, addressing the reviewer’s concern of accounting for important variables in the study.

### Outcome measures

The primary outcome of interest in this study is Postoperative Gastrointestinal Dysfunction (POGD), a significant postoperative complication affecting patients’ recuperation and quality of life post-surgery. POGD encompasses a range of gastrointestinal symptoms experienced by patients following surgery. According to the consensus developed by the second Perioperative Quality Initiative, which congregated international experts to formulate a clear definition for POGD, this condition is recognized as a significant concern in both clinical and research domains [18]. Additionally, in the context of intestinal surgery, POGD, along with Postoperative Ileus (POI), is acknowledged for manifesting through symptoms like nausea, vomiting, abdominal distention, bloating, and constipation [19].

In line with these authoritative insights, POGD in this study is characterized based on a spectrum of clinical symptoms including nausea, vomiting, abdominal distention, prolonged inability to pass flatus or stool, and a sustained inability to tolerate oral intake. The occurrence of these symptoms serves as the criteria for identifying the incidence of POGD among the studied patient cohort. Our definition and characterization of POGD are grounded in established clinical guidelines and literature, ensuring a standardized approach to identifying and measuring this primary outcome. This standardized criterion facilitates a robust comparison of postoperative outcomes between the case and control groups, thereby contributing to the validity and reliability of the study findings.

### Ethical considerations

All procedures performed in this study involving human participants were in accordance with the ethical standards of the institutional research committee and with the 1964 Helsinki declaration and its later amendments or comparable ethical standards. The study was approved by the Institutional Review Board (IRB) of our institution, and given its retrospective nature, the need for informed consent was waived.

### Statistical analysis

Statistical Analysis encompassed various methodologies to ensure rigor in data interpretation. Data were categorized as continuous or categorical variables. Continuous variables were represented as mean ± standard deviation (SD) or median (interquartile range, IQR) based on their distribution normality, assessed using Shapiro-Wilk test, while categorical variables were expressed as number (percentage). For comparing groups, statistical tests were chosen based on data type; Student’s t-test was employed for normally distributed continuous data, Mann-Whitney U test for non-normally distributed data, and Chi-square test or Fisher’s exact test for categorical variables to compare proportions between the case and control groups. Univariate and multivariate logistic regression analyses were conducted to identify factors associated with postoperative gastrointestinal dysfunction (POGD), with potential confounders identified based on clinical expertise and existing literature. Multicollinearity among independent variables was assessed using the variance inflation factor (VIF), with a threshold of 10 to indicate significant multicollinearity, and was addressed through statistical adjustments or exclusion from the model. Clinically relevant variables and those with *P* <  0.05 in univariate analysis were integrated into the multivariate logistic regression model to control for potential confounders. A two-tailed *P* value of < 0.05 was considered statistically significant, and alongside *P* values, odds ratios (ORs) with 95% confidence intervals were reported to elucidate the strength and direction of associations. ORs depict the odds of POGD occurrence between groups based on specified risk factors, aiding in clinical interpretation. Missing data were tackled using multiple imputations to create a complete dataset for analysis, thus minimizing bias, chosen for its superiority in handling missing data compared to listwise deletion. A post-hoc power analysis was conducted to ascertain the adequacy of the sample size, ensuring reliable detection of significant associations. Sensitivity analyses were performed to evaluate the robustness of the findings against potential biases and assumptions, assessing the impact of missing data and the choice of statistical models on the outcomes. The comparison with existing literature and generalizability portion remained unchanged, providing a solid comparison and acknowledging the limitations concerning the generalizability of the findings. The implications of the findings and the need for further research were highlighted in the Future Research and Clinical Implementation portion, which also remained unchanged. All statistical evaluations were executed using SPSS version 25.0 (IBM, USA), owing to its widespread acceptance in the research community and compatibility with our dataset.

## Results

### Patient characteristics

Table 1 meticulously delineates the baseline attributes of the 281 patients who were diagnosed with gynecological malignant tumors. The table encapsulates a wide array of vital parameters, thereby providing a holistic overview of the patient demographics and clinical features pertinent to the study. The serum bilirubin levels, indicative of liver health, are presented with a mean value of 11.4 and a standard deviation (SD) of 4.3485, reflecting the variability within the patient cohort. Additionally, the sodium levels, pivotal for cellular function and metabolic activities, are represented with a median value of 138.19 and an interquartile range (IQR) of 136.07 to 140.01, denoting the middle 50% range of the data. Surgical and anesthesia time, crucial indicators of procedure complexity and patient tolerance, respectively, are elucidated with median values and IQR, as well as categorical distributions, providing a nuanced understanding of the surgical interventions. The Body Mass Index (BMI) data is meticulously categorized into four distinct groups, shedding light on the nutritional status and potential obesity-related risks among the patients. The anatomical considerations, including the presence of the greater omentum and pelvic lymph nodes, are detailed with precise frequencies, offering insights into the anatomical variations and potential implications on surgical outcomes.

**Table 1:** Descriptive statistics for characteristics of patients with gynecological malignant tumor

Characteristics	Overall
Serum_bilirubin, mean ± sd	11.4 ± 4.3485
Sodium, median (IQR)	138.19 (136.07, 140.01)
Surgical_time, median (IQR)	4.9542 (3.9688, 6.2681)
Anesthesia_time, n (%)	
5-10 h	186 (66.2%)
> 10 h	37 (13.2%)
< 5 h	58 (20.6%)
bmi_category, n (%)	
25.0–29.9	117 (41.6%)
18.5–24.9	120 (42.7%)
< 18.5	13 (4.6%)
> 30	31 (11%)
greater_omentum, n (%)	
Yes	36 (12.8%)
No	245 (87.2%)
pelvic_lymph_node, n (%)	
Yes	140 (49.8%)
No	141 (50.2%)

The table presents the descriptive statistics for various characteristics of patients with gynecological malignant tumors. The characteristics include serum bilirubin, sodium, surgical time, anesthesia time, BMI category, greater omentum, and pelvic lymph node

### Case-control comparison

Table 2 embarks on a comprehensive comparative analysis between the case and control groups, accentuating the clinically significant variables that potentially influence postoperative outcomes, specifically postoperative gastrointestinal dysfunction (POGD). The serum bilirubin levels, a critical marker for liver health and potential hemolysis, are distinctly higher in the case group with a mean value of 14.172 and SD of 4.0701, as opposed to the control group’s mean value of 9.6429 and SD of 3.5351, the difference being statistically significant (*p* <  0.001). The sodium levels, indispensable for cellular homeostasis, exhibit a lower median value in the case group (136.26 with an IQR of 135.2–137.63) compared to the control group (139.32 with an IQR of 137.7–140.75), the disparity being statistically significant (*p* <  0.001). The surgical time, reflective of the procedure’s complexity, was significantly longer in the case group with a mean value of 6.1547 and SD of 1.9426 hours, compared to the control group’s mean value of 4.5959 and SD of 1.5475 hours (*p* <  0.001). Moreover, the categorical distributions of anesthesia time, BMI categories, the presence of the greater omentum, and pelvic lymph nodes are delineated alongside the respective *p*-values, underlining the statistical significance and potential clinical implications of these variables. Through this comparative analysis, a profound understanding of the patient demographics and clinical variables influencing postoperative outcomes is achieved, aiding clinicians in making informed, evidence-based decisions.

**Table 2:** Comparison of characteristics between case and control groups in patients with gynecological malignant tumor

Characteristics	Case	Control	*P* value
n	109	172	
Serum_bilirubin, mean ± sd	14.172 ± 4.0701	9.6429 ± 3.5351	< 0.001
Sodium, median (IQR)	136.26 (135.2, 137.63)	139.32 (137.7, 140.75)	< 0.001
Surgical_time, mean ± sd	6.1547 ± 1.9426	4.5959 ± 1.5475	< 0.001
Anesthesia_time, n (%)			< 0.001
5-10 h	88 (31.3%)	98 (34.9%)	
> 10 h	17 (6%)	20 (7.1%)	
< 5 h	4 (1.4%)	54 (19.2%)	
Bmi_category, n (%)			0.048
25.0–29.9	45 (16%)	72 (25.6%)	
18.5–24.9	45 (16%)	75 (26.7%)	
< 18.5	8 (2.8%)	5 (1.8%)	
> 30	11 (3.9%)	20 (7.1%)	
Greater_omentum, n (%)			< 0.001
Yes	33 (11.7%)	3 (1.1%)	
No	76 (27%)	169 (60.1%)	
Pelvic_lymph_node, n (%)			< 0.001
Yes	93 (33.1%)	47 (16.7%)	
No	16 (5.7%)	125 (44.5%)	

The table includes the following characteristics: serum bilirubin levels, sodium levels, surgical time, anesthesia time, BMI categories, the presence of the greater omentum, and the presence of pelvic lymph nodes

### Factors related to postoperative dysfunction

Table 3 showcases outcomes from univariate and multivariate analyses, identifying factors associated with postoperative gastrointestinal dysfunction. In the univariate analysis, serum bilirubin showed an inverse relationship, suggesting higher levels lead to a decreased odds ratio (OR = 0.722, 95% CI = 0.662–0.788, *p* <  0.001). Sodium levels and surgical time also exhibited associations with the outcome. In the multivariate analysis, variables were chosen based on their univariate significance and clinical relevance. This approach aimed to control for potential confounding factors. After adjustments, serum bilirubin retained its significance (OR = 0.777, 95% CI = 0.683–0.884, *p* <  0.001). Elevated sodium levels might increase the odds of gastrointestinal dysfunction. Despite anesthesia time being evaluated in the univariate analysis, it was excluded from the multivariate analysis due to its non-significance. The presence or absence of the greater omentum and pelvic lymph nodes also exhibited significant associations with gastrointestinal dysfunction.

**Table 3:** Univariate and multivariate analysis for factors associated with postoperative gastrointestinal dysfunction in patients with gynecological malignant tumor

Characteristics	Total(N)	Univariate analysis		Multivariate analysis	
		Odds Ratio (95% CI)	*P* value	Odds Ratio (95% CI)	*P* value
Serum_bilirubin	281	0.722 (0.662–0.788)	**< 0.001**	0.777 (0.683–0.884)	**< 0.001**
Sodium	281	1.774 (1.535–2.050)	**< 0.001**	1.818 (1.434–2.305)	**< 0.001**
Surgical_time	281	0.598 (0.509–0.702)	**< 0.001**	0.517 (0.388–0.689)	**< 0.001**
Anesthesia_time	281				
5-10 h	186	Reference		Reference	
> 10 h	37	1.056 (0.521–2.144)	0.879	0.650 (0.201–2.102)	0.472
< 5 h	58	12.122 (4.218–34.837)	**< 0.001**	16.221 (3.019–87.152)	**0.001**
Bmi_category	281				
25.0–29.9	117	Reference			
18.5–24.9	120	1.042 (0.616–1.760)	0.879		
< 18.5	13	0.391 (0.120–1.268)	0.118		
> 30	31	1.136 (0.498–2.592)	0.761		
Greater_omentum	281				
Yes	36	Reference		Reference	
No	245	24.461 (7.279–82.194)	**< 0.001**	19.083 (3.386–107.547)	**< 0.001**
Pelvic_lymph_node	281				
Yes	140	Reference		Reference	
No	141	15.459 (8.253–28.955)	**< 0.001**	16.507 (6.021–45.250)	**< 0.001**

The table presents the results of univariate and multivariate analysis examining the association between various characteristics and the development of postoperative gastrointestinal dysfunction in patients diagnosed with gynecological malignant tumors

### Predictive performance evaluation

Table 4 evaluates the predictive accuracy of serum bilirubin, sodium, surgical time, and a combined model. The AUC values for serum bilirubin, sodium, and surgical time were 0.798, 0.817, and 0.734, respectively. The combined model showcased an impressive AUC of 0.917. The optimal cut-off values were identified as 12.263 for serum bilirubin, 138.13 for sodium, and 5.2141 for surgical time. The combined model’s threshold was 0.01474.

**Table 4:** Evaluation of predictive performance for serum bilirubin, sodium, surgical time, and combined model using auc, 95% ci, best threshold, specificity, and sensitivity

Parameters	AUC	95% CI	Best threshold	Specificity(%)	Sensitivity(%)
Serum_bilirubin	0.798	0.745–0.851	12.263	0.68807	0.77326
Sodium	0.817	0.767–0.868	138.13	0.83486	0.71512
Surgical_time	0.734	0.673–0.795	5.2141	0.66972	0.73256
Model	0.917	0.884–0.949	0.01474	0.79817	0.88372

The table presents the performance measures for serum bilirubin, sodium, surgical time and the combined model in predicting the outcome. AUC, best threshold, specificity (%), and sensitivity (%) are given. The values for the combined model are also provided for comparison

**Fig. 1:**
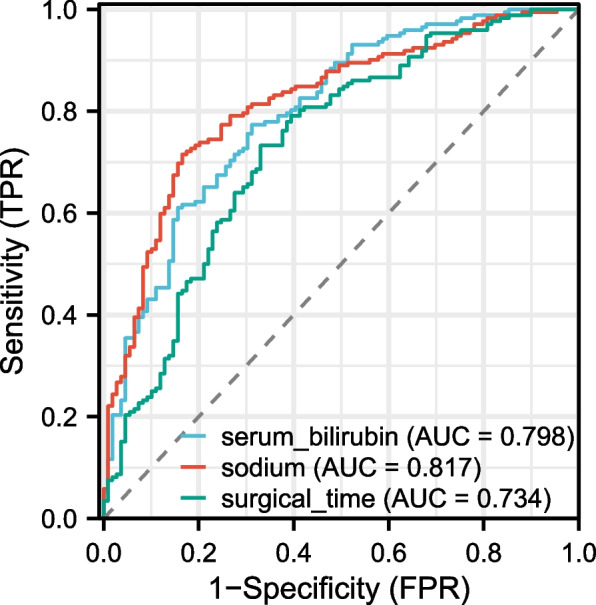
Receiver Operating Characteristic (ROC) Analysis of Serum Bilirubin, Sodium, Surgical Time for Predicting postoperative gastrointestinal dysfunction (POGD)

**Fig. 2:**
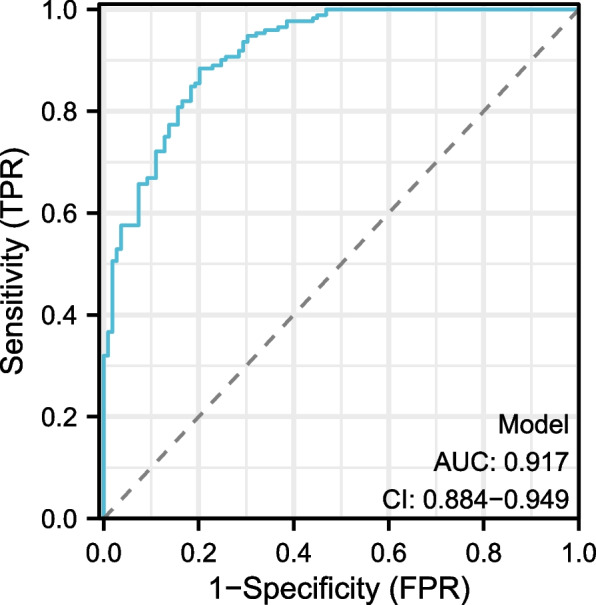
Receiver Operating Characteristic (ROC) Analysis of Combined Model for Predicting postoperative gastrointestinal dysfunction (POGD)

**Fig. 3:**
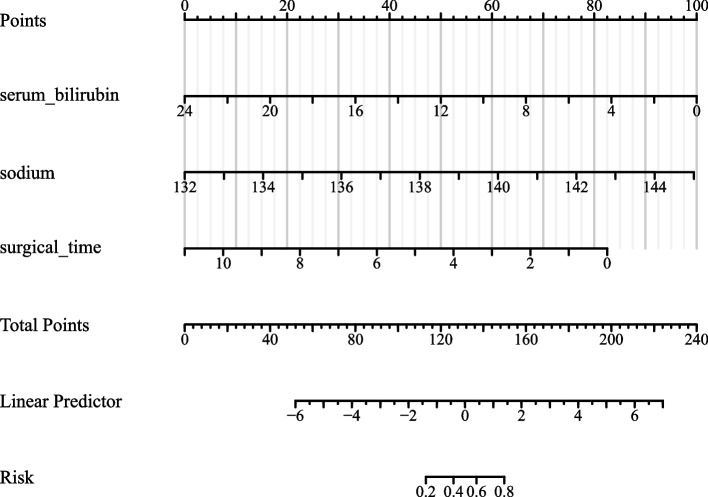
Nomogram that can predict postoperative gastrointestinal dysfunction (POGD)

Figures 1 and 2 visually represent these findings. Figure 1 provides an ROC curve for serum bilirubin, sodium, and surgical time. Figure 2 highlights the combined model’s ROC. Figure 3 introduces a straightforward nomogram based on the combined model. Clinicians can employ this nomogram to quickly estimate a patient’s risk of POGD, enhancing patient care decision-making. The robust AUC of 0.917 for our combined model emphasizes its predictive strength. However, for a more universal application, external validation across varied patient cohorts is essential.

## Discussion

Our study deepens the understanding of the associations between serum bilirubin levels, sodium levels, surgical time, anesthesia time, the presence of the greater omentum, pelvic lymph nodes, and postoperative gastrointestinal dysfunction (POGD) in patients with gynecological cancer.

### Comparative insights and biological mechanisms

Our study accentuates the multifactorial essence of Postoperative Gastrointestinal Dysfunction (POGD), mirroring the narrative presented by Chen et al. [20]. Their exploration of nutritional interventions in the context of gastric cancer underscores the significance of systemic support measures, particularly in enhancing immune function and nutritional status postoperatively. While they didn’t find an improvement in long-term prognosis, their findings are pivotal, highlighting the role of non-surgical interventions in patient recovery, an aspect our study extends by identifying physiological predictors like serum bilirubin levels.

Delving into the protective characteristics of elevated serum bilirubin, our study finds resonance with the work of Vítek et al. [21]. They established that individuals with higher serum bilirubin levels exhibited lower oxidative stress markers, implying bilirubin’s antioxidative capacity. This biochemical property, as suggested by their research, contributes to its role in countering inflammatory processes, a point that gains substantial backing from our findings regarding POGD’s multifaceted nature.

The concept of bilirubin acting beyond a metabolic by-product, potentially as a metabolic hormone, is an innovative view introduced by Creeden et al. [22]. They suggest that mild elevations in bilirubin levels could have systemic benefits, a hypothesis that our findings support. The physiological relevance of bilirubin that they propose offers an explanation for the protective trends observed in our study, suggesting a deeper biochemical interplay at work.

Contrasting these insights, our study observed nuances when compared with the findings of Wang et al. [23]. While they emphasized the influence of immediate postoperative dietary resumption in gastrointestinal function recovery, our research suggests that intrinsic factors like serum bilirubin levels also hold considerable sway in patient outcomes. This discrepancy indicates that recovery and protection mechanisms in postoperative contexts may extend beyond procedural factors, warranting comprehensive approaches in patient care.

Furthermore, our research complements the findings of So et al. [24] regarding the feasibility of surgical interventions in older gynecologic cancer patients. Our identification of protective physiological factors echoes the importance of considering comprehensive patient profiles, potentially influencing surgical outcomes and postoperative recovery.

In summation, our study not only underscores the protective nature of elevated serum bilirubin levels in the context of POGD but also reinforces the need for a holistic view of patient management, considering both intrinsic physiological factors and external interventions. The multifactorial complexity of POGD recovery elucidated herein calls for integrated strategies that encompass nutritional, biochemical, and surgical aspects, tailored to the individual’s physiological landscape.

### The role of surgical techniques in POGD risk

Surgical technique, as emphasized in [25], plays a multifaceted role in postoperative outcomes. The emerging vNOTES method, highlighted by [26], presents a less invasive approach, potentially reducing POGD risk. Yet, the surgeon’s expertise, as underscored by [27], significantly sways postoperative outcomes.

### Limitations and biases

In this study, we acknowledged several limitations and potential biases associated with our research design and execution. The retrospective nature of our medical record analysis confined us to available data recorded during standard care procedures, inherently introducing selection and information biases. Additionally, the sample from a single institution may not represent the broader population of patients with gynecological cancer, potentially limiting the generalizability of our findings. The retrospective characteristic of our study also precluded standardized outcome measurements, relying instead on clinical judgments and diagnostic practices of the time for each case. Despite utilizing logistic regression models to identify predictors of POGD, there might be unadjusted, unmeasured confounding variables affecting the outcomes. These could include genetic factors, underlying comorbidities, or lifestyle habits like diet and exercise, not extensively explored in our analysis. Considering these limitations, the findings of our study should be interpreted with caution. Future research would benefit from a multicentric, prospective study design with a larger, more diverse patient sample to validate our results and incorporate a more comprehensive set of variables, including genetic markers and detailed lifestyle factors, to further elucidate the complex dynamics influencing POGD in patients with gynecological cancer.

### Clinical implementation challenges

Challenges in assimilating our findings into clinical practice span from healthcare system adaptability to patient compliance. Strategies to circumvent these hurdles include continuous medical education, integrating findings into electronic health record systems, and structured patient counseling. The emphasis on predictive modeling’s role in clinical scenarios, as highlighted by [28], further underscores the need for such strategies.

## Conclusion

This research illuminates critical insights into the postoperative landscape for patients with gynecological cancer, identifying elevated serum bilirubin levels, specific sodium levels, and prolonged surgical time as significant predictors of Postoperative Gastrointestinal Dysfunction (POGD). Our evidence underscores serum bilirubin’s protective attribute, a novel revelation suggesting that monitoring and potentially manipulating this biochemical parameter could strategically mitigate POGD risk.

Moreover, this study paves the way for personalized perioperative care, advocating for a more nuanced approach that considers individual biochemical landscapes. While our findings mark a significant stride towards personalized healthcare paradigms in gynecological oncology, they also highlight the necessity for further expansive studies. Future research, especially multicentric and prospective studies, is indispensable to corroborate our findings, explore underlying mechanisms, and refine predictive models, ensuring their robustness and generalizability.

Overall, our study contributes important preliminary insights to the postoperative management of patients with gynecological cancer, suggesting that serum bilirubin levels could serve as a potential biochemical marker to predict POGD risk. These findings highlight the need for further research to validate the clinical implications and understand the underlying mechanisms better.

### Supplementary Information

**Additional file 1:** **Supplemental Fig. 1.** Comprehensive Visualization of Key Dataset Variables.

**Additional file 2.**

## Data Availability

The datasets analyzed during the current study are not publicly available due to privacy but are available from the corresponding author at a reasonable request.
