# Supplementary figures and images for: Predictive modeling of postoperative gastrointestinal dysfunction: the role of serum bilirubin, sodium levels, and surgical duration in gynecological cancer care
Source: BMC Womens Health. 2023 Nov 13;23:598. doi: 10.1186/s12905-023-02779-1 (PMC10644577; doi:10.1186/s12905-023-02779-1)

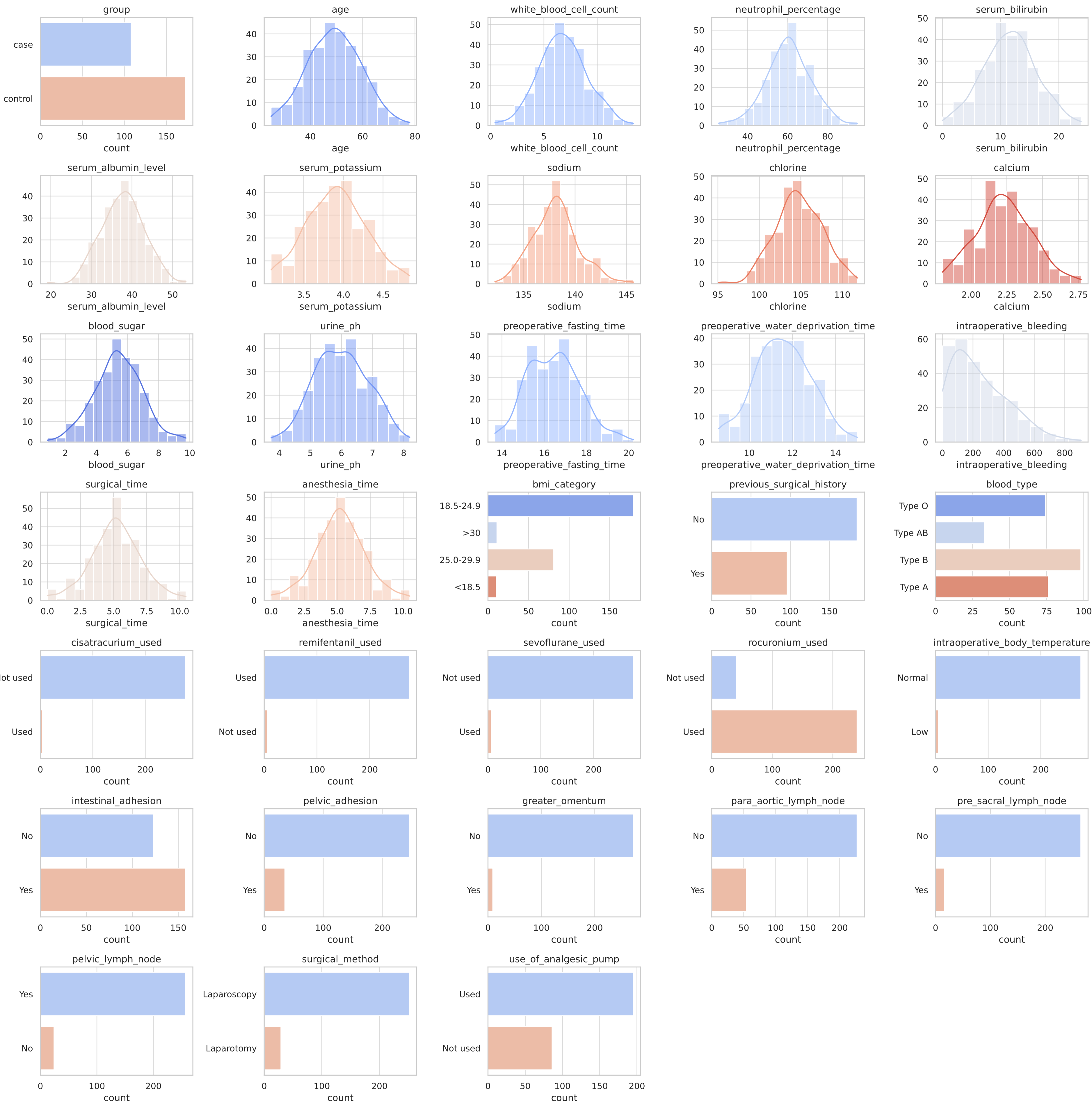

Supplement: Supplementary file 1 — Additional file 1: Supplemental Fig. 1. Comprehensive Visualization of Key Dataset Variables. [file 12905_2023_2779_MOESM1_ESM.pdf]
