# Supplementary material for: Predictive modeling of postoperative gastrointestinal dysfunction: the role of serum bilirubin, sodium levels, and surgical duration in gynecological cancer care
Source: BMC Womens Health. 2023 Nov 13;23:598. doi: 10.1186/s12905-023-02779-1 (PMC10644577; doi:10.1186/s12905-023-02779-1)
Supplement: Supplementary file 2 — Additional file 2. [file 12905_2023_2779_MOESM2_ESM.docx]

Supplementary Table 1: Comparison of Clinical Characteristics Between Case and Control Groups in Patients with Gynecological Malignant Tumors

| **Characteristics** | **case** | **control** | **P value** |
| --- | --- | --- | --- |
| n | 109 | 172 |  |
| age, mean ± sd | 49.472 ± 10.244 | 48.977 ± 10.155 | 0.692 |
| white_blood_cell_count, mean ± sd | 6.9199 ± 2.3255 | 6.7122 ± 2.1715 | 0.448 |
| neutrophil_percentage, mean ± sd | 59.551 ± 10.624 | 60.956 ± 11.55 | 0.308 |
| serum_bilirubin, mean ± sd | 14.172 ± 4.0701 | 9.6429 ± 3.5351 | < 0.001 |
| serum_albumin_level, mean ± sd | 37.817 ± 5.4552 | 37.779 ± 5.0674 | 0.953 |
| serum_potassium, mean ± sd | 3.9322 ± 0.35286 | 3.8931 ± 0.38737 | 0.396 |
| sodium, median (IQR) | 136.26 (135.2, 137.63) | 139.32 (137.7, 140.75) | < 0.001 |
| chlorine, mean ± sd | 105.01 ± 2.6228 | 104.64 ± 2.9481 | 0.286 |
| calcium, mean ± sd | 2.2579 ± 0.19553 | 2.2126 ± 0.19535 | 0.060 |
| blood_sugar, mean ± sd | 5.3971 ± 1.604 | 5.4089 ± 1.4915 | 0.950 |
| urine_ph, mean ± sd | 6.1201 ± 0.8441 | 5.9254 ± 0.87325 | 0.067 |
| preoperative_fasting_time, mean ± sd | 16.375 ± 1.1238 | 16.485 ± 1.3086 | 0.469 |
| preoperative_water_deprivation_time, mean ± sd | 11.614 ± 1.2225 | 11.517 ± 1.3296 | 0.540 |
| intraoperative_bleeding, median (IQR) | 207.1 (91.861, 401.97) | 211.69 (104.84, 357.29) | 0.810 |
| surgical_time, mean ± sd | 5.177 ± 1.8821 | 5.1937 ± 1.929 | 0.943 |
| surgical_time, mean ± sd | 6.1547 ± 1.9426 | 4.5959 ± 1.5475 | < 0.001 |
| anesthesia_time, n (%) |  |  | < 0.001 |
| 5-10h | 88 (31.3%) | 98 (34.9%) |  |
| >10h | 17 (6%) | 20 (7.1%) |  |
| <5h | 4 (1.4%) | 54 (19.2%) |  |
| bmi_category, n (%) |  |  | 0.048 |
| 25.0-29.9 | 45 (16%) | 72 (25.6%) |  |
| 18.5-24.9 | 45 (16%) | 75 (26.7%) |  |
| <18.5 | 8 (2.8%) | 5 (1.8%) |  |
| >30 | 11 (3.9%) | 20 (7.1%) |  |
| previous_surgical_history, n (%) |  |  | 0.817 |
| No | 72 (25.6%) | 113 (40.2%) |  |
| Yes | 36 (12.8%) | 60 (21.4%) |  |
| blood_type, n (%) |  |  | 0.455 |
| Type O | 31 (11%) | 43 (15.3%) |  |
| Type AB | 12 (4.3%) | 21 (7.5%) |  |
| Type B | 32 (11.4%) | 66 (23.5%) |  |
| Type A | 33 (11.7%) | 43 (15.3%) |  |

Supplementary Table 1 (Continued): Comparison of Additional Characteristics Between Case and Control Groups in Patients with Gynecological Malignant Tumors

| **Characteristics** | **case** | **control** | **P value** |
| --- | --- | --- | --- |
| n | 109 | 172 |  |
| cisatracurium_used, n (%) |  |  | 0.969 |
| Not used | 107 (38.1%) | 170 (60.5%) |  |
| Used | 1 (0.4%) | 3 (1.1%) |  |
| remifentanil_used, n (%) |  |  | 1.000 |
| Used | 106 (37.7%) | 169 (60.1%) |  |
| Not used | 2 (0.7%) | 4 (1.4%) |  |
| sevoflurane_used, n (%) |  |  | 1.000 |
| Not used | 106 (37.7%) | 169 (60.1%) |  |
| Used | 2 (0.7%) | 4 (1.4%) |  |
| rocuronium_used, n (%) |  |  | 0.666 |
| Not used | 17 (6%) | 24 (8.5%) |  |
| Used | 91 (32.4%) | 149 (53%) |  |
| intraoperative_body_temperature, n (%) |  |  | 0.696 |
| Normal | 107 (38.1%) | 169 (60.1%) |  |
| Low | 1 (0.4%) | 4 (1.4%) |  |
| intestinal_adhesion, n (%) |  |  | 0.291 |
| No | 43 (15.3%) | 80 (28.5%) |  |
| Yes | 65 (23.1%) | 93 (33.1%) |  |
| pelvic_adhesion, n (%) |  |  | 0.590 |
| No | 96 (34.2%) | 150 (53.4%) |  |
| Yes | 12 (4.3%) | 23 (8.2%) |  |
| greater_omentum, n (%) |  |  | < 0.001 |
| Yes | 33 (11.7%) | 3 (1.1%) |  |
| No | 76 (27%) | 169 (60.1%) |  |
| para_aortic_lymph_node, n (%) |  |  | 0.698 |
| No | 86 (30.6%) | 141 (50.2%) |  |
| Yes | 22 (7.8%) | 32 (11.4%) |  |
| pre_sacral_lymph_node, n (%) |  |  | 0.327 |
| No | 100 (35.6%) | 165 (58.7%) |  |
| Yes | 8 (2.8%) | 8 (2.8%) |  |
| pelvic_lymph_node, n (%) |  |  | < 0.001 |
| Yes | 93 (33.1%) | 47 (16.7%) |  |
| No | 16 (5.7%) | 125 (44.5%) |  |
| surgical_method, n (%) |  |  | 0.387 |
| Laparoscopy | 99 (35.2%) | 153 (54.4%) |  |
| Laparotomy | 9 (3.2%) | 20 (7.1%) |  |
| use_of_analgesic_pump, n (%) |  |  | 0.779 |
| Used | 76 (27%) | 119 (42.3%) |  |
| Not used | 32 (11.4%) | 54 (19.2%) |  |
